# Inhibition of IL-6 Signaling Pathway by Curcumin in Uterine Decidual Cells

**DOI:** 10.1371/journal.pone.0125627

**Published:** 2015-05-11

**Authors:** Y. Sangeeta Devi, Majesta DeVine, Justin DeKuiper, Susan Ferguson, Asgerally T. Fazleabas

**Affiliations:** Department of Obstetrics, Gynecology and Reproductive Biology, College of Human Medicine, Michigan State University, Grand Rapids, MI, United States of America; Queen's University, CANADA

## Abstract

IL-6 is a multifunctional pro-inflammatory cytokine and has been implicated in many gestational disorders including preterm birth. Currently, there are no appropriate therapeutic interventions available to circumvent inflammatory-mediated gestational disorders. Therefore, the goal of this study was to identify a safe and effective pharmacological compound to counterbalance inflammatory responses in the uterus. Curcumin, a naturally-occuring polyphenolic compound, has been widely used in alternative medicine to treat inflammatory diseases. However, the anti-inflammatory effect of curcumin has not been explored in uterine decidual cells, a major source of IL-6. Therefore, we examined the effect of curcumin on IL-6 expression using two types of uterine decidual cells 1) HuF cells, primary human fibroblast cells obtained from the decidua parietalis; 2) UIII cells, a rodent non-transformed decidual cell line. Curcumin treatment completely abrogated the expression of IL-1β-induced IL-6 in these cells. Curcumin also strongly inhibited the expression of gp130, a critical molecule in IL-6 signaling, whereas expression of IL-6R and sIL-6R was not affected. Curcumin also inhibited phosphorylation and nuclear localization of STAT3, a well-known downstream mediator of IL-6 signaling. Furthermore, curcumin attenuated IL-1β-induced IL-6 promoter reporter activity suggesting transcriptional regulation. To further understand whether NF-ҡB is involved in this inhibition, we examined the effect of curcumin on the expression of p50 and p65 subunits of NF-ҡB in decidual cells. Expression of IL-1β-induced p50 mRNA was repressed by curcumin while p65 mRNA was not affected. However, curcumin treatment dramatically inhibited both p50 and p65 protein levels and prevented its nuclear localization. This effect is at least partly mediated through the deactivation of IKK, since IL-1β-induced IKKα/β phosphorylation is decreased upon curcumin treatment. Our results not only revealed molecular mechanisms underlying curcumin action in uterine decidual cells but also suggest that this compound may have therapeutic potential for the prevention of inflammation-mediated preterm birth and other gestational disorders.

## Introduction

Interleukin-6 (IL-6) is a multifunctional cytokine with pivotal roles in the inflammatory response in many tissues. It mediates its action by binding to a transmembrane cognate receptor, IL-6R, resulting in homodimerization of a signal-transducing glycoprotein, gp130 [1,2]. This triggers a complex intracellular cascade that results in a concerted transcriptional increase of genes with critical roles in inflammation. Expression of IL-6 is induced during inflammation, infection, trauma, and stress as a consequence of induction by stimuli including IL-1, Tumor Necrosis Factors (TNF), Lipopolysaccharide (LPS) and Toll-Like receptor ligands [3–5]. Elevated IL-6 has been implicated in various gestational disorders such as unexplained infertility, recurrent miscarriage, preeclampsia and preterm delivery [6]. Particularly, there is compelling evidence for involvement of IL-6 in parturition and attests for a strong correlation of increased IL-6 levels and preterm birth [6]. Expression of IL-6 is very low or undetectable at mid-gestation in normal pregnancy, but is induced in the uterus upon infection [7,8]. Increased concentrations of IL-6 are found in the cervical, amniotic and vaginal fluid of women delivering preterm [8–10]. Genetic association studies demonstrated that a single nucleotide polymorphism in the promoter region of the IL-6 gene is associated with increased risk of preterm birth [11,12]. In another study, polymorphisms in the IL-6 and IL-6R gene that correlate with amniotic fluid IL-6 concentration are associated with the incidence of preterm delivery [13]. A recent study examining various inflammatory markers in preterm delivery reported that elevated IL-6 displayed the strongest association with spontaneous preterm delivery at <35 weeks as well as spontaneous preterm delivery accompanied by chorioamnionitis [10]. Furthermore, IL-6 knockout mice have delayed parturition and are protected against low dose LPS-induced preterm birth [14]. The role of IL-6 as a molecular signal for termination of pregnancy is further supported by the fact that IL-6 regulates several genes involved in labor. IL-6 up-regulates the production of the prostaglandins PGE2 and PGF2α, and the PGF2α receptor in cells from the human uterus *in vitro* [15,16].

Although there is an overwhelming amount of data supporting the involvement of IL-6 and inflammatory pathway in preterm labor and other gestational disorders, to date, there is no ideal therapeutic intervention targeting this pathway. The most common interventions recommended to prevent or treat preterm labor, such as bed rest, tocolytic or antibiotic treatment, and cervical cerclage have proved to be of little or no benefit [17]. Steroid based anti-inflammatory agents are not ideal for treatment of preterm birth due to severe side effects including adverse mental health in childhood and adolescence when exposed prenatally [18]. Recently, progesterone supplementation for women with a previous preterm delivery or a short cervix has shown some promise of effectiveness [17,19]. Even if it proves efficacious for these specific at-risk populations, progesterone treatment would prevent only a small proportion of preterm births. Therefore, there is an urgent need to identify potential therapeutic targets and inhibitory molecules to intervene in inflammation-mediated preterm birth and other gestational disorders. Curcumin, a naturally occuring polyphenolic compound isolated from the spice turmeric, has been used widely in traditional medicine to treat inflammatory and infectious diseases [20]. This compound became an attractive candidate in modern medicine due to its ability to interact with many signaling molecules while being considered safe for human health [21]. There is growing evidence that curcumin has a broad range of molecular targets, including inflammatory cytokines [22]. Although a large body of literature has supported a protective role of curcumin in various inflammatory diseases, its effect and therapeutic potential for inflammation related pregnancy complications have not been examined thoroughly.

Previous reports have shown that decidual cells, which are derived from maternal endometrial stroma, are a major source of elevated IL-6 levels during pregnancy complications [8,23]. IL-6 is highly induced in decidual explants in response to bacterial lipopolysaccharide (LPS) or IL-1β [24,25]. These findings suggest that IL-6 produced by uterine decidual cells in response to inflammatory stimuli contributes to the pathophysiology of preterm labor and other gestational disorders. Therefore, supplementation of anti-inflammatory reagents that can inhibit IL-6 expression and other cytokines may be beneficial in preventing inflammation-mediated gestational disorders. Keeping this in mind, we investigated the effect of curcumin on the inflammation-induced IL-6 and associated signaling molecules in human and rodent uterine stromal/decidual cells. We show that curcumin is a potent inhibitor of IL-6 in uterine decidual/stromal cells and suggest the involvement of IKK/NF-κB pathway in this inhibition.

## Materials and Methods

### Human placenta

Human term placentae with attached fetal membranes were obtained through the Michigan State University’s Center for Women’s Health Research Female Reproductive Tract Biorepository. All samples were obtained with written informed consent from women who delivered healthy, singleton infants at term (>37 weeks gestation) with normal vaginal delivery. The protocols were approved by the Institutional Review Boards, Human Research Protection Program at Michigan State University (East Lansing, MI) and Spectrum Health Systems (Grand Rapids, MI). Small pieces of tissues were fixed from several parts of placenta and fetal membrane. Frozen sections of preterm placenta/fetal membrane slides were provided by the NIH Placental Bank at University of California, San Francisco (UCSF), funded under NIH HD055764-06. These samples were collected with protocols approved by Institutional Review Board, Committee on Human Research (CHR) at UCSF, and with written informed consent from women who delivered singleton infants with spontaneous preterm labor. Tissue samples used in study were preterm delivery at 34–29 weeks. The clinical information of these samples is summarized in the S1 Table.

### Cell culture

Primary Uterine Decidual/Stromal Cell culture: Human uterine fibroblasts (HuF) cells were isolated from the decidua parietalis dissected from the placental membrane after normal vaginal delivery. Tissue was obtained after informed consent under a protocol approved by the Institutional Review Boards at both the Michigan State University and Spectrum Health. Isolation procedure was performed as described previously [26]. Briefly, after trypsinization, dissociated cells were maintained and propagated in RPMI 1640 media supplemented with 10% fetal bovine serum (FBS). From a single placenta, we isolated approximately 1.5 × 10^8^ cells. The cells isolated from each individual patient represented a proliferating population of nondifferentiated stromal fibroblasts and were always used between passages 2–4. UIII cells were cultured as described previously [27]. The generation and characterization of UIII cells has been described previously [28,29]. All experiments were conducted using 3–4 different HuF cell isolations or UIII cell cultures, and assays were performed in duplicates. For transient transfection, cells were grown at 60–80% confluency in RPMI media supplemented with 4% charcoal dextran treated FBS (CDS-FBS) in six-well plates. Cells were transfected using Lipofectamine 2000 (Life technologies, Carlsbad, CA) or Effectine (Qiagen, Valencia, CA) according to the manufacturer’s protocol. For treatments, cells were cultured in RPMI media supplemented with 4% CDS-FBS and treated with vehicle, recombinant human IL-1β (10ng/ml, Sigma Chemical, St. Louis, MO), curcumin (1–40μM, Sigma Chemical, St. Louis, MO), IL-1β (10ng/ml)+ curcumin (1–40μm). After 24 hr, culture media was collected and cells were washed and frozen at -80°C for later used.

### sIL-6R ELISA

Huf cells were cultured and treated with vehicle (control) or IL-1β (10ng/ml) in the presence or the absence of curcumin (30μM) for 24hr. The culture media from Huf cell cultures were assessed for sIL-6R concentrations using commercially available enzyme-linked immunosorbent assay (ELISA) according to the manufacturer's instructions (Life Technologies, Carlsbad, USA). All data were corrected for total protein and expressed pg per mg protein. The protein content of the spent medium was determined using the BCA protein assay (Pierce, Rockford, USA), using bovine serum albumin as a reference standard. The calculated inter-assay and intra-assay coefficients of variation were all <10%.

### qPCR and RT-PCR

Total RNA was extracted from treated HuF and UIII cells using TRIzol reagent (Life Technologies, Carlsbad, CA) according to the manufacturer's instructions. One microgram of total RNA was reverse transcribed using iScript cDNA kit according to the manufacturer's instructions (BioRad, Hercules, CA). The resulting cDNA was then diluted to a total volume of 100 μl with sterile H_2_O. Each qPCR consisted of 2.5 μl of diluted RT product, 1X SYBR Green PCR Master Mix (PE Applied Biosystems, Foster City, CA), and 50 nm forward and reverse primers. The reactions were carried out on an ABI PRISM 7700 sequence detection system (PE Applied Biosystems) for 40 cycles (95°C for 15 s and 60°C for 1 min) after the initial 10 min incubation at 95°C. The primer sets used for HuF cells were: IL-6 (5’- TCA ATG AGG AGA CTT GCC TGG TGA-3’ and 5’- TCA TCT GCA CAG CTC TGG CTT GTT-3’), IL-6R (5’- AGT ATT CCC AGG AGT CCC AGA AGT-3’ and 5’-TTG CTG AAC TTG CTC CCG ACA CTA-3’), gp130 (5’- TCA CAA TCC TGT GGA TCT GGG CAA-3’ and 5’- CAG CCT CCA TGC CAA CTG TTT CAA-3’), p65 NF-κB (5’- ATC TGC CGA GTG AAC CGA AAC TCT-3’ and 5’- CTG GTC CCG TGA AAT ACA CCT CAA -3’), p50 NF-κB (5’-AGG ATG AAG GAG TTG TGC CTG GAA-3’ and 5’- TCA GCC AGC TGT TTC ATG TCT CCT-3’) and RPS17 (5’-ATG AAG CGA ATT CAG AGA GGC CCA-3’ and 5’- CAA GGC TGA GAC CTC AGG AAC ATA-3’). The primer sets used for UIII cells were: IL-6 (5’-TGGGACTGATGTTGTTGACAGCCA-3’ and 5’-AGCCTCCGSCTTGTGAAGTGGTAT-3’), L19 (5’-TGGAGCACATCCACAAACTGAAGG-3’ and 5’-CGCTTTCGTGCTTCCTTGGTC-TTA-3’). The fold change in expression of each gene was calculated using the ΔΔ*C*_t_ method, with the ribosomal protein RPS17 or L19 mRNA as an internal control. For RT-PCR, Conditions for each template were optimized so that signals were in the linear range of detection. The PCR products with DNA loading buffer were then separated by gel electrophoresis on a 0.7% agarose gel.

### Reporter Assay

IL-6 promoter reporter constructs were a gift from Dr. Ernesto Canalis (Department of Research, Saint Francis Hospital and Medical Centre, Hartford, USA) with the permission of Dr. George Fey (University of Erlangen-Nuremberg, Erlangen, Germany). The constructs were transfected and cells were treated as described previously in “Cell Culture” section. Luciferase activity driven by the IL-6 promoter construct was measured by combining the lysate with firefly luciferase assay substrate (Promega, Madison, WI) according to manufacturer's protocol. Luciferase activity was normalized to respective firefly Renilla activity.

### Western Blot Analysis

For Western blots, 25μg of protein was resolved on SDS-PAGE, transferred to PVDF membranes, and blocked for nonspecific binding. The membranes were then incubated with the appropriate primary antibodies overnight at 4°C. After a series of washes, the blots were incubated with a secondary antibody linked to horseradish peroxidase for 1 hr. The bands were detected by using an enhanced chemiluminescence system. Primary antibodies used were rabbit polyclonal anti-phospho-pSTAT3 (EMD Millipore, Billerica, MA), rabbit polyclonal anti-IL-6 (Abcam Inc., Cambridge, MA), rabbit polyclonal anti-phospho-IKKα/β (Cell Signaling Technology, Inc. Danvers, MA), and mouse monoclonal anti-β-Actin (Sigma Chemical, St. Louis, MO). The secondary antibodies used were goat anti-rabbit or goat anti-mouse IgG (Jackson ImmunoResearch Laboratories, West Grove, PA).

### Immunocytochemistry

HuF and UIII cells were grown for 24 hr in RPMI and M199 medium supplemented with 4% CDT-FBS on Lab-Tek chamber slides (Nalge Nunc International, Rochester, NY). The cells were cultured with IL-1β, IL-1β+curcumin, or vehicle for 24 hr and processed for immuno-cytochemistry as described previously [30]. Primary antibodies used were rabbit polyclonal anti-p50 and goat polyclonal anti- p65 NF-κB (Santa Cruz Biotechnology, Inc., Dallas, Tx), whereas Cy3-conjugated or Cy2 conjugated donkey anti-rabbit IgG (Jackson ImmunoResearch Laboratories, West Grove, PA) were used as secondary antibodies. The slides were mounted in Vectashield medium (Vector Laboratories, Inc., Burlingame, CA) containing a counterstain for DAPI and were observed with a Nikon Eclipse Ni microscope equipped with Nikon DS-Qi1Mc and DS-Fi2 digital cameras.

### Immunohistochemistry

For term placenta, paraffin-embedded sections were subjected to the avidin-biotin-peroxidase complex method using a Vectastain ABC kit (Vector Laboratories, Inc., Burlingame, CA) as described previously with some modifications [30]. Briefly, formalin-fixed, paraffin-embedded sections (5μm) were deparaffinized, hydrated, and blocked with 5% normal donkey serum. The slides were then incubated overnight at 4°C with a polyclonal antibody to IL-6 (Abcam Inc., Cambridge, MA) or a mouse monoclonal antibody to IGFBP-1 at 1:100. The slides were then incubated with corresponding secondary biotinylated goat IgG according to the manufacturer’s instructions (Vectastain ABC kit; Vector Laboratories, Burlingame, CA). Peroxidase activity was developed with Nova Red or DAB solution (Vector Laboratories, Inc., Burlingame, CA). For preterm placenta, frozen sections were fixed in pre-cooled methanol at -20°C for 10 min. Sections were processed for immunohistochemistry as described for term placenta. Tissue sections from at least 3 different placentas were used for each group.

### Statistical analysis

The data were examined by one-way analysis of variance followed by the Tukey-Kramer Multiple Comparisons Test using InState software (GraphPad Software Inc., San Diego, CA). The values were considered statistically significant at *p* < 0.05 (*), *p* < 0.01 (**) and *p* < 0.001 (***).

## Results

### Expression of IL-6 in decidual cells in human placenta

**Fig 1:**
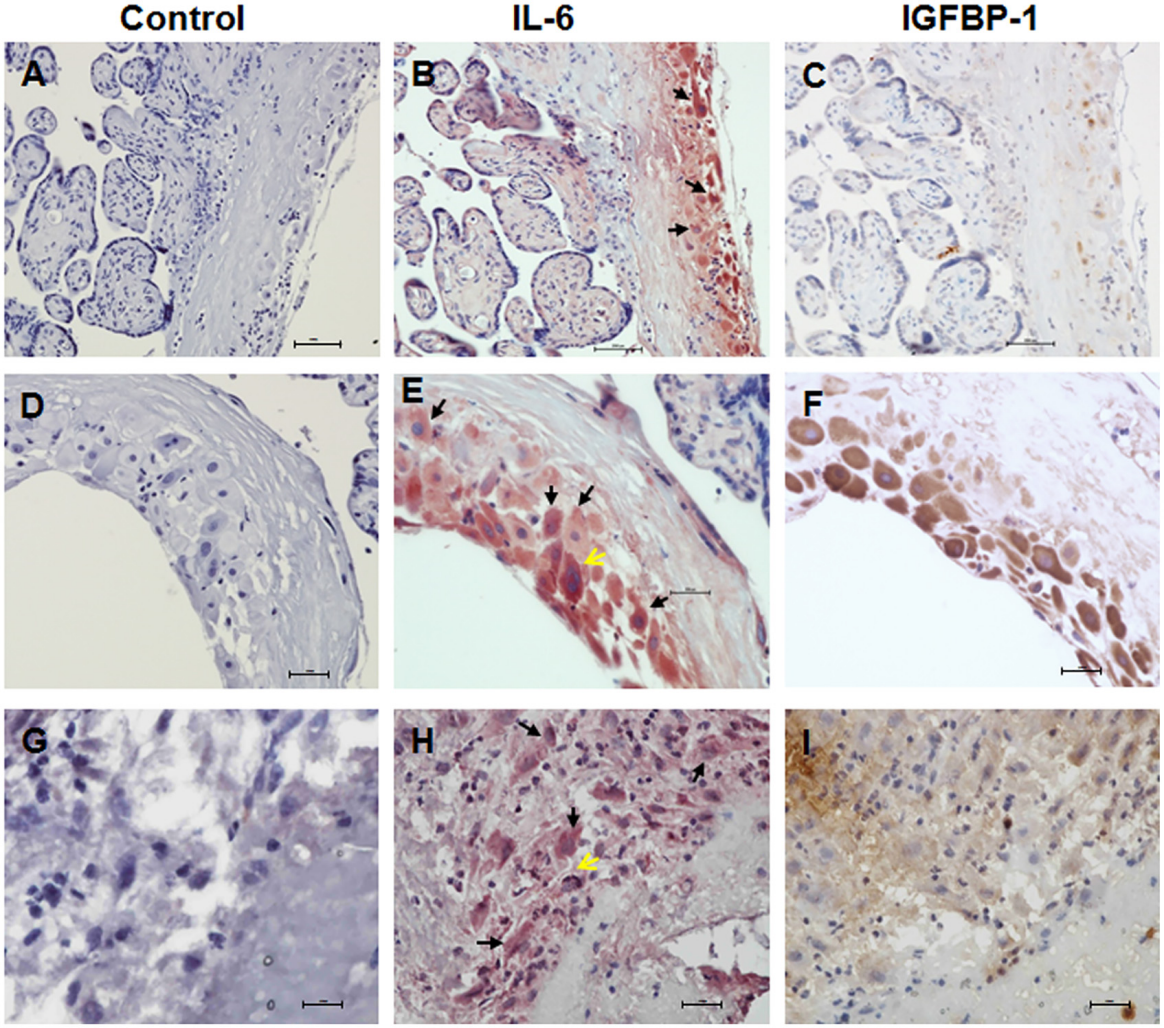
Expression of IL-6 in decidual cells in human placenta. Paraffin embedded sections of human term placenta (A-F) and frozen sections of idiopathic preterm placenta (G-I) with some attached fetal membrane were analyzed for IL-6 or IGFBP-1 expression by immunohistochemistry. Decidual cells were identified by morphological analysis or IGFBP-1 staining. (A) Term placenta/fetal membrane, control immunostaining. (B) Term placenta/fetal membrane, IL-6 expression is shown in red staining, decidual cells (arrows). (C) Term placenta/fetal membrane, IGFBP-1 expression is shown in brown staining. A-C: adjacent slides of same placenta/fetal membrane. (D) Term placenta/fetal membrane, control immunostaining. (E) Term placenta/fetal membrane, IL-6 expression is shown in red staining, decidual cells (arrows), trophoblast cells (yellow arrows). (F) Term placenta/fetal membrane, IGFBP-1 expression is shown in brown staining. D-F are adjacent slides of another term placenta/fetal membrane at higher magnification. (G) Preterm placenta/fetal membrane, control immunostaining. (H) Preterm placenta/fetal membrane, IL-6 expression is shown in red staining, decidual cells (arrows), trophoblast cells (yellow arrow). (I) Preterm placenta/fetal membrane, IGFBP-1 expression is shown in brown staining. G-I are adjacent slides of idiopathic spontaneous preterm placenta/fetal membrane. At least 3 different placentas were used in each group. Shown in the figure are representative tissue slides. Original magnification: A-C, 20X; D-I, 40X.

Expression of IL-6 was examined by immunohistochemistry in the serial sections of human placenta and fetal membrane from term (Fig 1A–1F) and preterm deliveries (Fig 1G–1I). Intense IL-6 staining was observed in the decidual cells of term (Fig 1B and 1E) and preterm placenta (Fig 1H) as indicated by arrows, whereas very little or no staining was observed in the villi. We also found IL-6 staining in trophoblast cells (yellow arrows). The presence of decidua was confirmed by their distinct morphology, as well as by immunostaining with IGFBP-1 in adjacent slides (Fig 1C, 1F and 1I). Control adjacent slides were processed similarly without the primary antibody and no staining was detected (Fig 1A, 1D and 1G).

### Responsiveness of HuF and UIII cells to inflammatory stimuli

**Fig 2:**
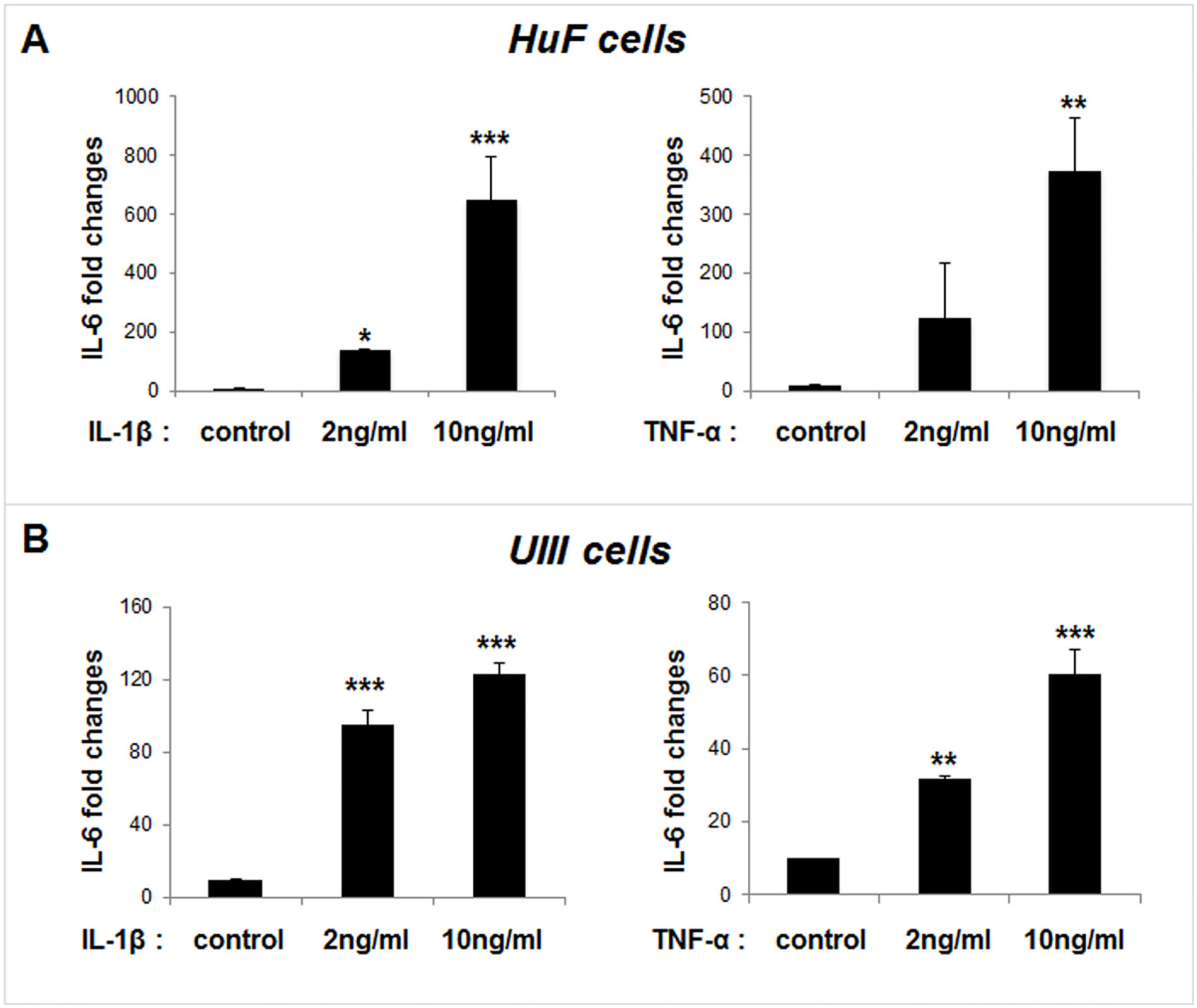
Responsiveness of HuF and UIII cells to inflammatory stimuli. Primary human uterine fibroblasts (HuF) cells were isolated from the decidua parietalis of placenta as described in materials and methods. UIII cells, a rat uterine stromal cell line that decidualizes in culture, were cultured as described previously [27]. Cells were treated with vehicle, or different doses of IL-1β or TNF-α for 24 hr. Total mRNA was analyzed for IL-6 expression in HuF cells (A), and UIII cells (B) by qPCR. RPS17 or L19 expression was used as control. The values are expressed as the means ± S.E. (*n* = 3). *, *p* < 0.05; **, *p* < 0.01; ***, *p* < 0.001.

To examine if HuF and UIII cells are responsive to inflammatory stimuli, cells were treated with different doses of IL-1β (2ng/ml and 10ng/ml) or TNF-α (10ng/ml and 20ng/ml) for 24 hr. As shown in Fig 2, expression of IL-6 was stimulated by IL-1β in a dose dependent manner in both HuF and UIII cells. Treatment with 2ng/ml of IL-1β shows a 10-fold increase in IL-6 expression while 10ng/ml of IL-1β produced ≥ 40-fold increase in HuF cells (Fig 2A, left panel). IL-1β also produced significant stimulation of IL-6 expression in UIII cells, namely ≥ 8-fold and ≥ 11-fold increase respectively by treatment with 2ng/ml and 10ng/ml of IL-1β (Fig 2B, left panel). Similar response was produced by TNF-α in both HuF and UIII cells although the fold increase was slightly lower as compared to IL-1β (Fig 2A and 2B, right panels).

### Dose dependent inhibition of IL-1β induced IL-6 expression by curcumin in decidual/stromal cells

**Fig 3:**
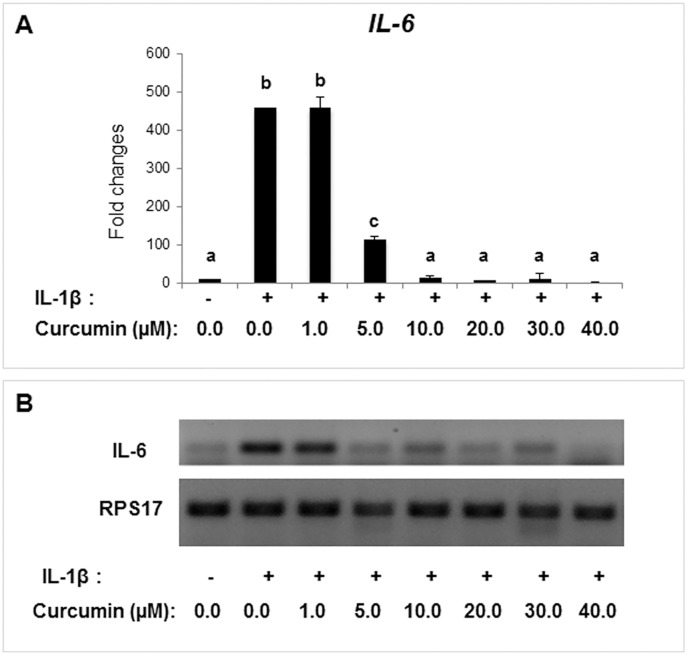
Dose dependent inhibition of IL-1β-stimulated IL-6 expression by curcumin. HuF cells were treated with vehicle (control), IL-1β (10ng/ml), or with combinations of IL-1β (10ng/ml) and different doses of curcumin (0–40μM) for 24 hr. Total mRNA was analyzed for IL-6 expression by qPCR and RT- PCR. RPS17 expression was used as control. The values are expressed as the means ± S.E. (*n* = 3). ab, *p* < 0.001; bc, *p* < 0.001; ac, *p* < 0.01.

To examine whether curcumin has any effect on inflammation-induced IL-6 in decidual/stromal cells, HuF and UIII cells were treated with IL-1β (10ng/ml) in the presence or the absence of different doses of curcumin for 24 hr. Expression of IL-6 was markedly stimulated by IL-1β but curcumin treatment inhibited this stimulation in a dose dependent manner in HuF cells (Fig 3). While 1μM Curcumin had no effect, 5μM—30μM curcumin completely abrogated IL-1β-induced IL-6 expression without causing any cell toxicity. 40μM of curcumin also inhibited IL-6 expression; however, there was some cell toxicity at this concentration. Curcumin (30μM) completely inhibited expression of IL-1β-induced IL-6 expression in UIII cells also (S1 Fig). Therefore, further studies were performed with 30μM curcumin.

### Regulation of IL-6 associated signaling molecules by curcumin in decidual/stromal cells

**Fig 4:**
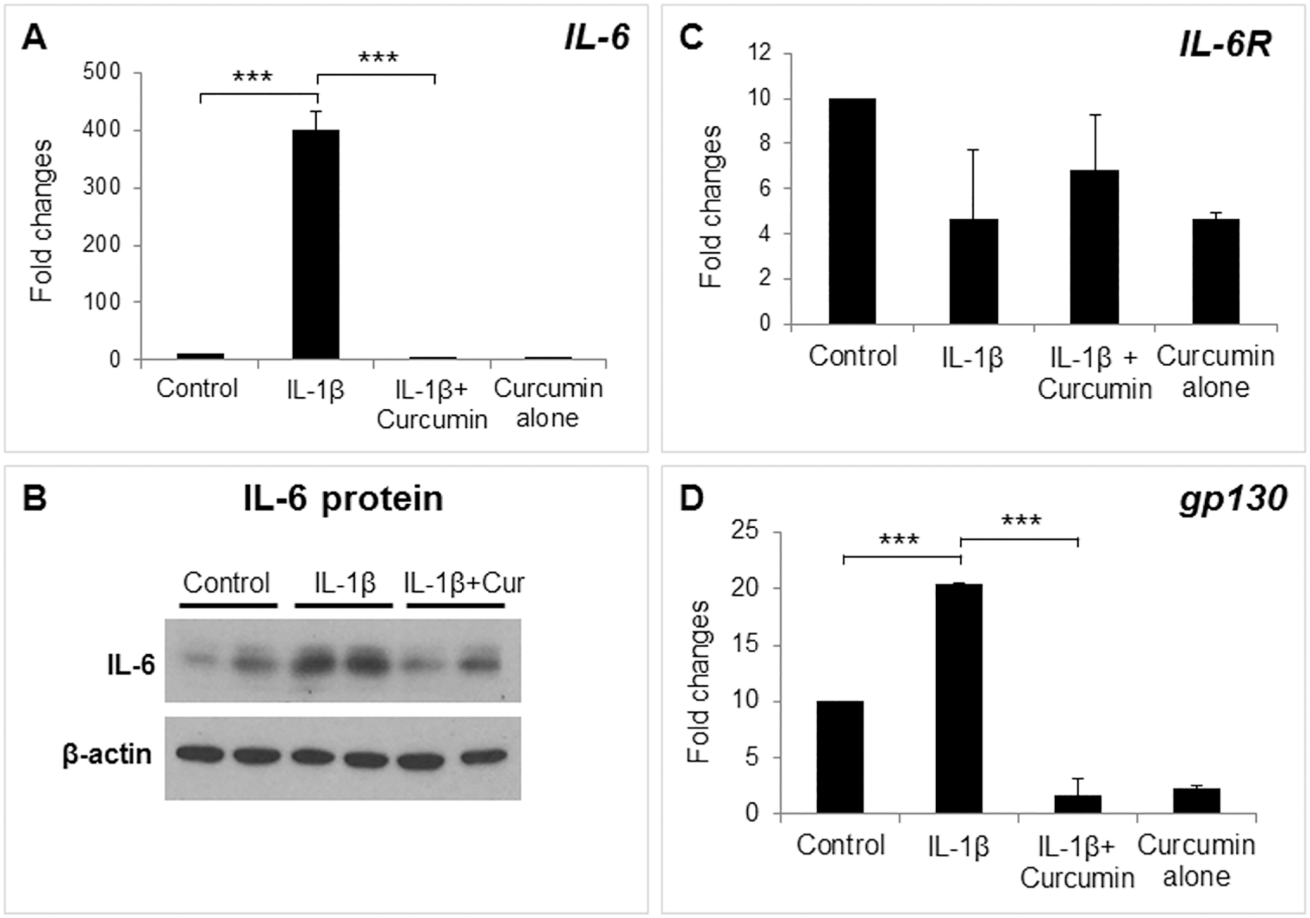
Regulation of IL-6 signaling pathway molecules by curcumin. HuF cells were treated with either vehicle (control), IL-1β (10ng/ml), or with a combination of IL-1β (10ng/ml) and curcumin (30μM), or curcumin alone (30μM). Total mRNA was analyzed for IL-6 (A), IL-6R (C) and gp130 (D) expression by qPCR. (B) Expression of IL-6 at protein level was examined by Western blot analysis. The values are expressed as the means ± S.E. (*n* = 3). ***, *p* < 0.001.

To examine whether curcumin affected expression of IL-6 signaling components, IL-6R, and gp130, HuF cells were treated with IL-1β with or without curcumin. As expected, curcumin treatment completely abrogated IL-1β-stimulated IL-6 mRNA transcription (Fig 4A) as well as protein levels (Fig 4B). Expression of IL-6R mRNA was not affected by curcumin (Fig 4C), whereas gp130 expression was significantly downregulated (Fig 4D). Treatment with curcumin alone had no significant effect on any of the genes analyzed as compared to respective controls. To examine the effect of curcumin on the IL-6 trans-signal, we measured the release of the soluble form of IL-6R (sIL-6R) in Huf cell media. We found no change in the concentration of sIL-6R upon IL-1β or curcumin treatment (S2 Fig)

### Transcriptional repression of IL-6 and inhibition of NF-κB transcription factors by curcumin

**Fig 5:**
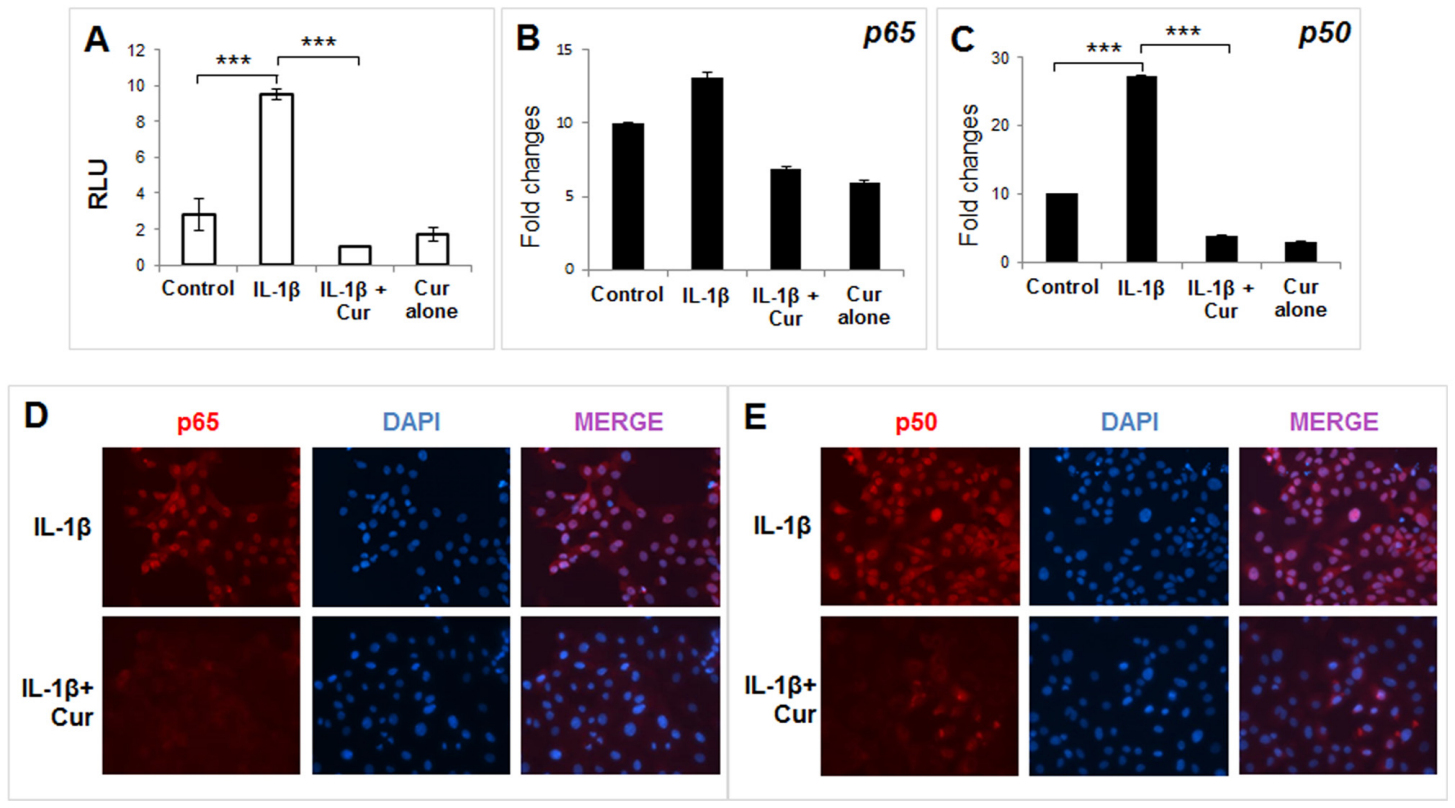
Transcriptional repression of IL-6 and regulation of NF-κB by curcumin. UIII and HuF cells were transfected with an IL-6 promoter reporter construct spanning -276 to +20 bp region. Cells were then treated with vehicle (control), IL-1β (10ng/ml), or with a combination of IL-1β (10ng/ml) and 30μM curcumin, or curcumin alone for 24hr. (A) IL-6 promoter activity was measured by dual luciferase assay in UIII cell lysates and normalized to Renilla. (B, C) Expression of p65 and p50 subunits of NF-κB was analyzed in HuF cells by qPCR. The values are expressed as the means ± S.E. (*n* = 3). ***, *p* < 0.001. (D, E) UIII cells were treated with IL-1β (10ng/ml), or with a combination of IL-1β and curcumin (30μM) for 24 hr. Cells were fixed in paraformaldehyde and processed for immunocytochemistry to analyze p65 and p50 NF-κB cellular localization. *Red*, p65/p50; *blue*, DAPI.

To examine whether repression of IL-1β-induced IL-6 expression by curcumin is at the level of transcription, UIII cells were transfected with an IL-6 promoter reporter construct spanning -276 to +20 bp [31]. The activity of IL-6 promoter reporter construct was stimulated significantly by IL-1β-induced activity, whereas this stimulation was completely abrogated by curcumin (Fig 5A). This promoter construct spanning -276 to +20 bp contains a putative NF-κB. To examine whether the inhibition of IL-6 gene expression involves inhibition of NF-κB, expression of p65 (RelA) and p50 subunits of NF-κB were analyzed at mRNA levels. Expression of p65 mRNA remained unchanged in response to IL-1β alone or in combination with curcumin (Fig 5B); whereas IL-1β treatment caused a 2.5-fold increase in the expression of p50 NF-κB, which was completely prevented by co-treatment with curcumin (Fig 5C). We also examined whether curcumin affects protein concentration and nuclear localization of p50 and p65 NF-κB in UIII cells. As shown in Fig 5D and 5E, both p50 as well as p65 subunits were localized in the nucleus of IL-1β treated cells, whereas curcumin treatment dramatically inhibited the expression of NF-κB and any remaining protein was excluded from the nucleus.

### Inhibition of IKK activation by curcumin in decidual/stromal cells

**Fig 6:**
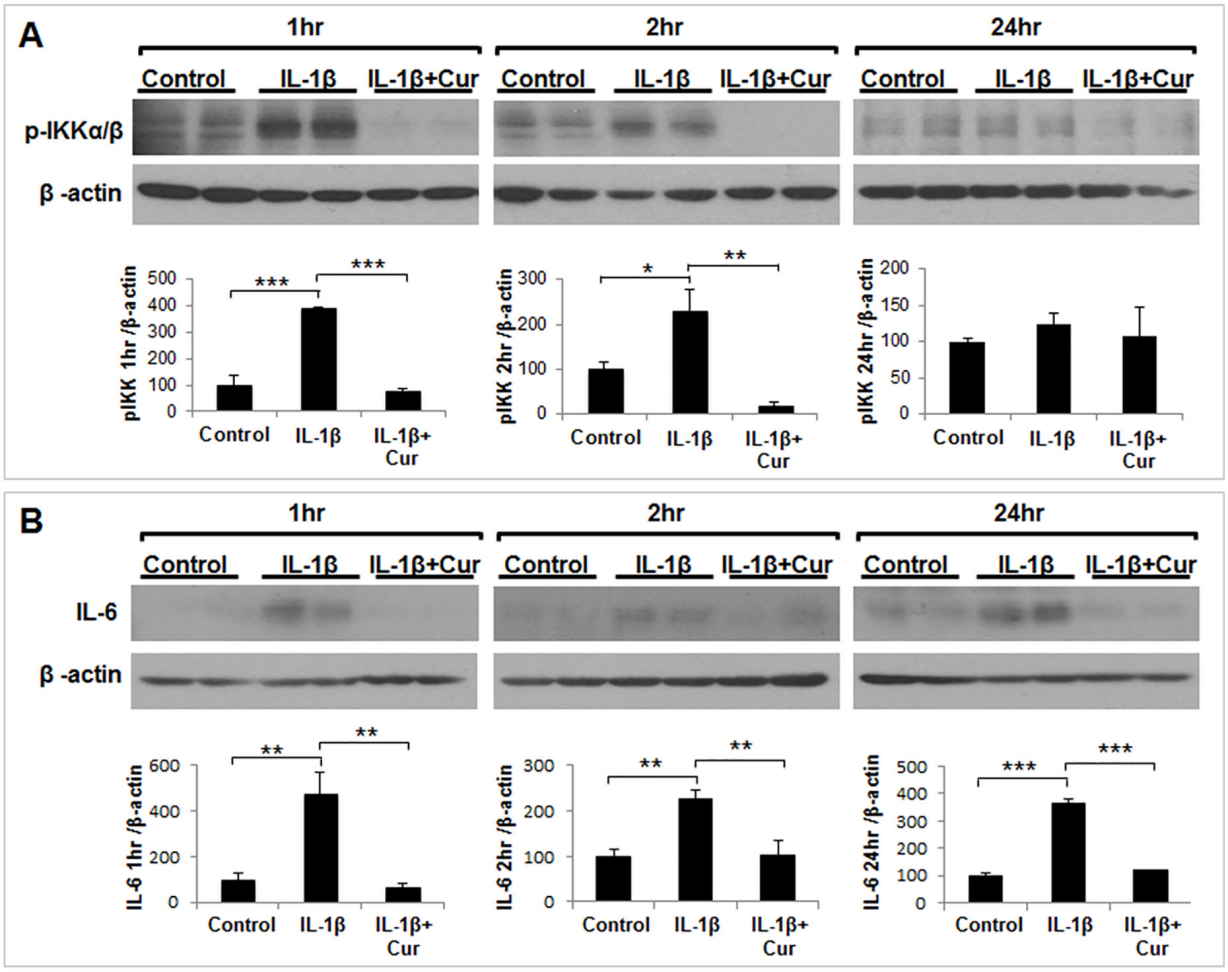
Inhibition of IKK activation by curcumin in decidual/stromal cells. HuF cells were treated with either vehicle (control), IL-1β (10ng/ml), or with a combination of IL-1β and curcumin (30μM), or curcumin alone for different time points (1–24 hr). (A) Phosphorylation of IKKα/β (p-IKKα/β) was analyzed by Western blot using phospho-specific antibody and β-actin was used as loading control (*upper panel*). The band intensity of p-IKKα/β was plotted against β-actin and normalized to percentage of control (*lower panel*). (B) Expression of IL-6 was analyzed by Western blot and β-actin was used as loading control (*upper panel*). The band intensity of IL-6 was plotted against β-actin and normalized to percentage of control (*lower panel*). The values are expressed as the means ± S.E. (*n* = 3). *, *p* < 0.05; **, *p* < 0.01; ***, *p* < 0.001.

To investigate further the signaling pathway that regulates activation/inhibition of NF-κB, we examined phosphorylation/activation of IKK in response to IL-1β with or without curcumin in HuF cells (Fig 6A). Treatment with IL-1β for 1 hr induced >300% increase in IKKα/β phosphorylation as compared to control whereas a 200% increase in phosphorylation of IKKα/β was observed at 2 hr treatment with IL-1β. Co-treatment with curcumin completely inhibited activation of IKK at both time points. The increased or decreased phosphorylation of IKK was directly correlated with a change in IL-6 expression at 1 hr and 2 hr (Fig 6B). Interestingly, phosphorylation of IKKα/β was not affected by IL-1β at 24 hr, although IL-6 expression was induced by IL-1β at this time point (Fig 6B) and NF-κB remained activated in the nucleus (Fig 5D and 5E).

### Curcumin mediated inhibition of IL-6 downstream signaling

**Fig 7:**
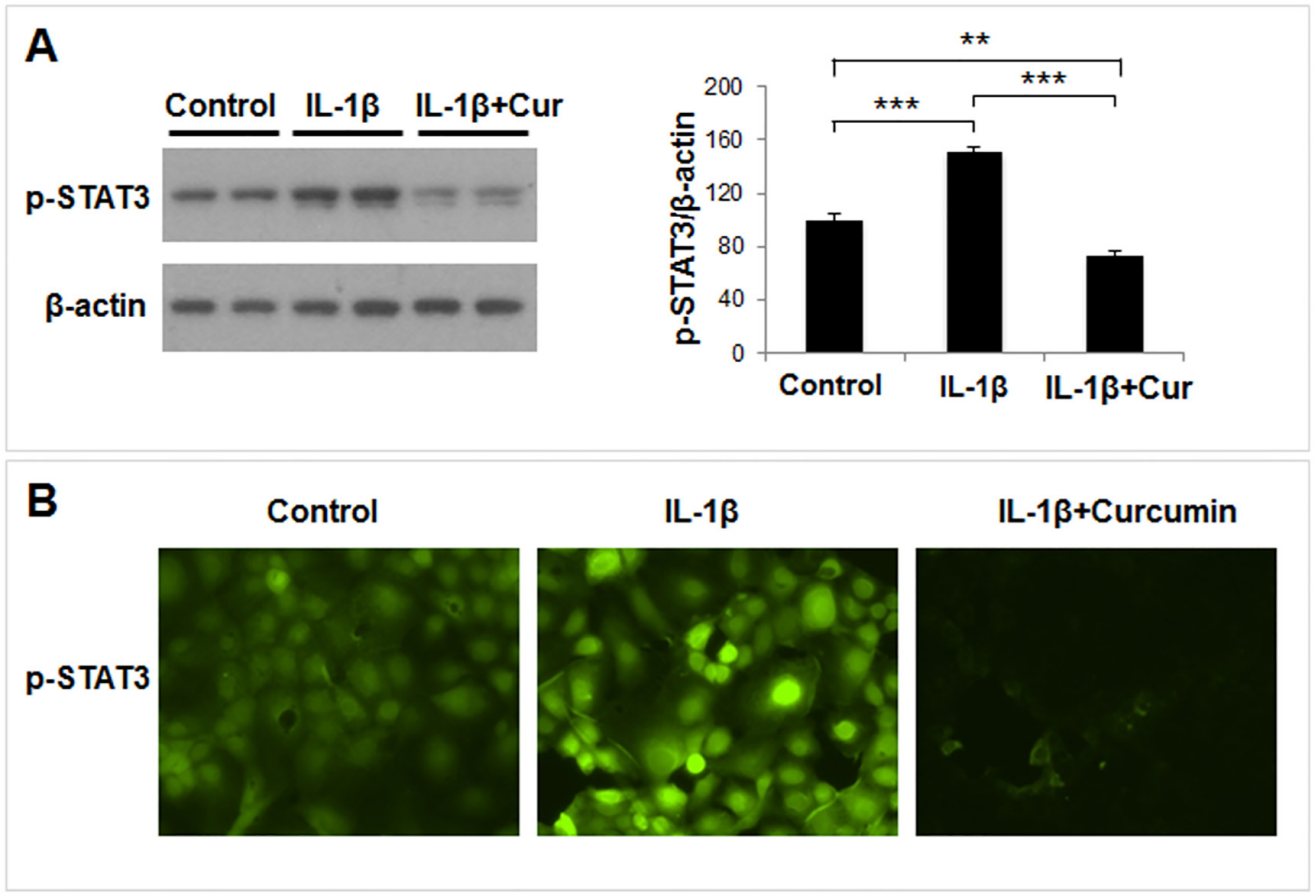
Curcumin mediated inhibition of IL-6 downstream signaling. HuF cells were treated with either vehicle (control), IL-1β (10ng/ml), or with a combination of IL-1β (10ng/ml) and curcumin (30μM), or curcumin alone (30μM) for 24 hr. (A) Phosphorylation of STAT3 (p-STAT3) was analyzed by Western blot using a phospho-specific antibody (*left panel*). β-actin was used as loading control. The band intensity of p-STAT3 was plotted against β-actin and normalized to percentage of control (*right panel*). The values are expressed as the means ± S.E. (*n* = 3). *, *p* < 0.05; **, *p* < 0.01; ***, *p* < 0.001. (B) UIII cells were treated with IL-1β (10ng/ml), or with a combination of IL-1β (10ng/ml) and curcumin (30μM) for 24 hr. Cells were fixed in paraformaldehyde and processed for immunocytochemistry to analyze p-STAT3 (*green*) cellular localization.

Next, we examined activation status of STAT3, a well-known downstream signaling mediator of IL-6 in UIII and HuF cells. Phosphorylation of STAT3 was significantly stimulated by IL-1β, whereas curcumin treatment completely inhibited IL-1β-induced STAT3 phosphorylation as well as constitutive (control) STAT3 phosphorylation (Fig 7A). Furthermore, we demonstrated that IL-1β treatment caused nuclear localization of phosphorylated (p) STAT3 in UIII cells. In contrast, cells treated with a combination of IL-1β and curcumin show very little or no nuclear pSTAT3.

## Discussion

The results of this investigation established that IL-6 is highly expressed in the decidual cells of placenta obtained from normal term delivery as well as idiopathic preterm delivery. Using a rodent decidual cell line and primary human decidual/stromal cells, our results clearly demonstrated that curcumin potently inhibited expression of IL-6 and its associated signaling molecule gp130 as well as downstream mediator, STAT3. Furthermore, our results suggest that the inhibition of IL-6 by curcumin is mediated at the level of transcription, and that repression of the IKK/NF-κB signaling pathway may be involved in this process.

Over 60% of preterm deliveries are unexplained and idiopathic in nature [32]. It is believed that these are associated with a (sub)clinical inflammatory response in the maternal and/or fetal tissues [32]. Increased concentrations of IL-6 in the cervical, amniotic, and vaginal fluid each reliably predict preterm birth associated with infection [8–10]. Elevated cervical IL-6 levels are also found in asymptotic women delivering preterm [33]. Interestingly, IL-6 levels are also upregulated in amniotic fluid and uterine tissues in term labor without any sign of infection [34,35]. Decidual cell, the predominant endometrial stromal cell type during pregnancy, has been proposed as a major source of IL-6 [36]. Our observation that intense IL-6 staining is detected in the decidual cells of placenta obtained from normal spontaneous term delivery supported the previous notion that IL-6 is associated with labor and decidua is a major source of this cytokine. Strikingly, we found strong expression of IL-6 in the decidual cells of placenta from idiopathic spontaneous preterm labor. Previous studies demonstrated that spontaneous labor is characterized by a genomic signature of acute inflammation including elevation of IL-6 in the chorioamniotic membranes (containing decidua) even in the absence of infection [37], while that of cytokine profile in maternal circulation remains unchanged [37,38]. This led Romero and colleagues to suggest that the inflammatory process is localized to the gestational tissues, and specifically to the membranes which contains decidua [39]. Another study also demonstrated that increases in IL-6 in cervicovaginal fluid and amniotic fluid but not in plasma are strongly associated with spontaneous preterm birth in asymptomatic women, suggesting that inflammation at the maternal-fetal interface, rather than systemic inflammation, may play a major role in the etiology of such spontaneous preterm births [40]. Our finding that IL-6 is expressed in the decidua of idiopathic spontaneous preterm labor is consistent with these observations and indicates that regardless of the presence of infection or gestational age, upregulation of IL-6 in gestational tissue is associated with spontaneous labor.

We found that human primary decidual/stromal cells (HuF) isolated from term placenta exhibited a profound upregulation of IL-6 in response to either IL-1β or TNF-α, two classic mediators of inflammation at the maternal-fetal interface. We also found a similar effect of IL-1β and TNF-α on IL-6 expression in UIII cells, a well characterized rodent decidual cell line [41]. These results are in agreement with previous findings that IL-6 production is induced by IL-1 and TNF-α treatment in cultured human decidual cells [23,25]. Thus, we established that HuF and UIII cells maintain signature cytokine expression profile in response to inflammatory insults. These cell culture systems enable us to examine the anti-inflammatory effect of pharmacological reagents. Since many anti-inflammatory reagents such as non-steroidal anti-inflammatory drugs (NSAID) or glucocorticoids carry adverse outcomes during pregnancy [18,42,43], we explored the potential of an alternative and natural compound, curcumin, as an anti-inflammatory reagent in our decidual/stromal cell culture model. Review of literature suggests that curcumin may be beneficial against many inflammatory diseases including obesity, diabetes, cardiovascular and neurodegenerative diseases, cerebral edema, allergy, bronchial asthma, inflammatory bowel disease, rheumatoid arthritis, renal ischemia, psoriasis, scleroderma, acquired immunodeficiency syndrome, and certain types of cancers [Rev in [44]]. Curcumin has been shown to inhibit IL-6 in various cell types and in many of these disease conditions [21,44,45]. However, the literature regarding the effect of curcumin on pregnancy and gestational disorders is scarce. Excitingly, in this study we clearly demonstrate that curcumin inhibits IL-1β-induced IL-6 expression in decidual/stromal cells. This finding is consistent with a recent study in which curcumin was shown to inhibit LPS, a known inducer of IL-1 production [46,47], -stimulated IL-6 release by human placenta and foetal membrane explants [48]. Since, no study has examined the mechanism of curcumin action in gestational tissues; we investigated the effect of this compound on both upstream and dowstream IL-6 signaling pathways. First, we evaluated the effect of curcumin on the IL-6 signaling components, IL-6R, sIL-6R and gp130. Previous studies have reported that decidua is a target of IL-6 action and expression of IL-6R and gp130 was shown in human as well as rodent decidual cells [1,7]. Our results demonstrated that IL-6R and sIL-6R, as well as their signaling component, gp130, were expressed in HuF and UIII cells, although expression of only gp130 was affected by curcumin. To our knowledge, this is the first report showing a direct effect of curcumin in attenuation of IL-1β-induced gp130 expression in any cell type. Our results suggest that curcumin inhibits the IL-1β-stimulated IL-6 signaling pathway in decidual/stromal cells by not only decreasing availability of IL-6, but also that of gp130. It appears that curcumin could potentially affect both classical and trans-signal IL-6 signaling pathways in decidual cells, since gp130 is involved in both the pathways [2]. Although gp130 and its dimer partners possess no intrinsic tyrosine kinase domain, the dimerization of gp130 leads to activation of associated cytoplasmic tyrosine kinases and subsequent modification of transcription factors [49]. This led us to examine the effect of curcumin on the activation/phosphorylation status of STAT3, which is a signature downstream transcription factor activated by IL-6 signaling. We found an increase in phosphorylation and nuclear localization of STAT3 in decidual/stromal cells upon IL-1β stimulation in concurrence with an increase in IL-6 expression, which was completely abrogated by curcumin treatment. Previous reports have demonstrated that curcumin can abrogate constitutive phosphorylation of STAT3 in various cancer cell lines [50–52]. Indeed, we also found that curcumin treatment reduced STAT3 phosphorylation below that of basal (control) STAT3 phosphorylation in decidual cells. These results indicate that curcumin may have a direct effect on constitutive STAT3 phosphorylation in addition to mediating its affect by means of decreasing IL-6 expression.

IL-6 expression can be regulated at multiple levels [53]. Our results clearly established that curcumin-mediated inhibition of IL-1β-induced IL-6 expression in decidual/stromal cells involves transcriptional mechanisms. The IL-6 promoter contains multiple regulatory transcription factor elements [54]. Among these, NF-κB site (-73 to -64) in the IL-6 promoter was proposed to be necessary for IL-1 induced expression [55]. NF-κB is a family of transcription factors that are functional as dimeric complexes and is a known target of curcumin [56]. Therefore, we examined the effect of curcumin on the IL-1β-stimulated expression of p50 and p65 (RelA), two subunits of NF-κB that are involved in canonical NF-κB signaling pathway. Curcumin effectively attenuated IL-1β-induced p50 mRNA expression, whereas neither IL-1β nor curcumin had any effect on the p65 mRNA expression. Curcumin alone had no apparent effect on basal mRNA expression of either p65 or p50 NF-κB. However, at the protein level, not only does curcumin exclude nuclear localization of both p65 and p50 induced by IL-1β but it also inhibits their expression profoundly. These results suggest that curcumin mediates its inhibitory effect by attenuating an NF-κB activation pathway and/or NF-κB protein stability. Since IKKα/β plays critical role in the activation of NF-κB, we examined the effect of curcumin on IL-1β-induced IKKα/β activation. We found that phosphorylation of IKKα/β was markedly increased at 1 hr and 2 hr of IL-1β treatment in decidual/stromal cells. This was associated with activation of NF-κB and an increase in IL-6 expression. This IL-1β-stimulated phosphorylation of IKK was completely abrogated by curcumin, which was associated with a sharp drop in IL-6 expression. These results suggest that curcumin-mediated inhibition of IL-6 is, at least partly, dependent on the deactivation of IKK/NF-κB signaling pathway. Interestingly, at 24hr, NF-κB remained activated/nuclear-localized and expression of IL-6 was strongly induced by IL-1β, although IKKα/β phosphorylation was reduced to basal level at this time point. The discrepancy may be due to a delay in the synthesis of IκBα (the inhibitor of NF-κB) or the interaction of IκBα and NF-κB.

In addition to control of NF-κB activity by an upstream mechanism, NF-κB has been shown to be the target of nuclear inhibition by protein degradation. COMMD1 and PDLIM2 are two of the proteins that have been shown to interact with NF-κB, leading to its ubiquitination and proteasomal degradation in the nucleus [57]. Our finding that curcumin not only repressed NF-κB activation but also profoundly inhibited its expression suggests that nuclear protein degradation may be involved in the curcumin-mediated inhibition of p65 and p50 NF-κB. It is an intriguing possibility that curcumin stimulates expression of COMMD1/PDLIM2 and/or induced its interaction with NF-κB. Further investigations are required to delineate alternative mechanisms in the curcumin-mediated inhibition of NF-κB/IL-6 signaling pathways in decidual/stromal cells. Taken together, our results have established that curcumin is a potent inhibitor of NF-κB/IL-6 signaling pathway in uterine decidual/stromal cells. Since intrauterine inflammation plays a major role in preterm birth and IL-6 is a pivotal molecule in this process, supplementation of curcumin may be beneficial for the prevention of human preterm labor and ultimately preterm birth.

## Supporting Information

S1 Fig Inhibition of IL-1β-stimulated IL-6 expression by curcumin in UIII cells. UIII cells were treated with vehicle (control), IL-1β (10ng/ml), or with a combination of IL-1β (10ng/ml) and curcumin (30μM) for 24hr. Expression of IL-6 was analyzed by qPCR (A) and Western blot (B). RPS17 expression was used as control for qPCR and β-actin was used as loading control for Western blot analysis. The values are expressed as the means ± S.E. (*n* = 3). ***, *p* < 0.001.
(TIF)

S2 Fig Effect of IL-1β and curcumin on sIL-6R release by Huf cells. HuF cells were treated with either vehicle (control), IL-1β (10ng/ml), or with a combination of IL-1β (10ng/ml) and curcumin (30μM) for 24hr. The culture media were assessed for sIL-6R concentrations using a ELISA kit. The values were corrected for total protein and expressed as pg/mg protein and expressed as the means ± S.E. (*n* = 3).
(TIF)

S1 Table Available clinical information of mothers/placentas. (DOCX)
