# Supplementary figures and images for: Inhibition of IL-6 Signaling Pathway by Curcumin in Uterine Decidual Cells
Source: PLoS One. 2015 May 11;10(5):e0125627. doi: 10.1371/journal.pone.0125627 (PMC4427355; doi:10.1371/journal.pone.0125627)

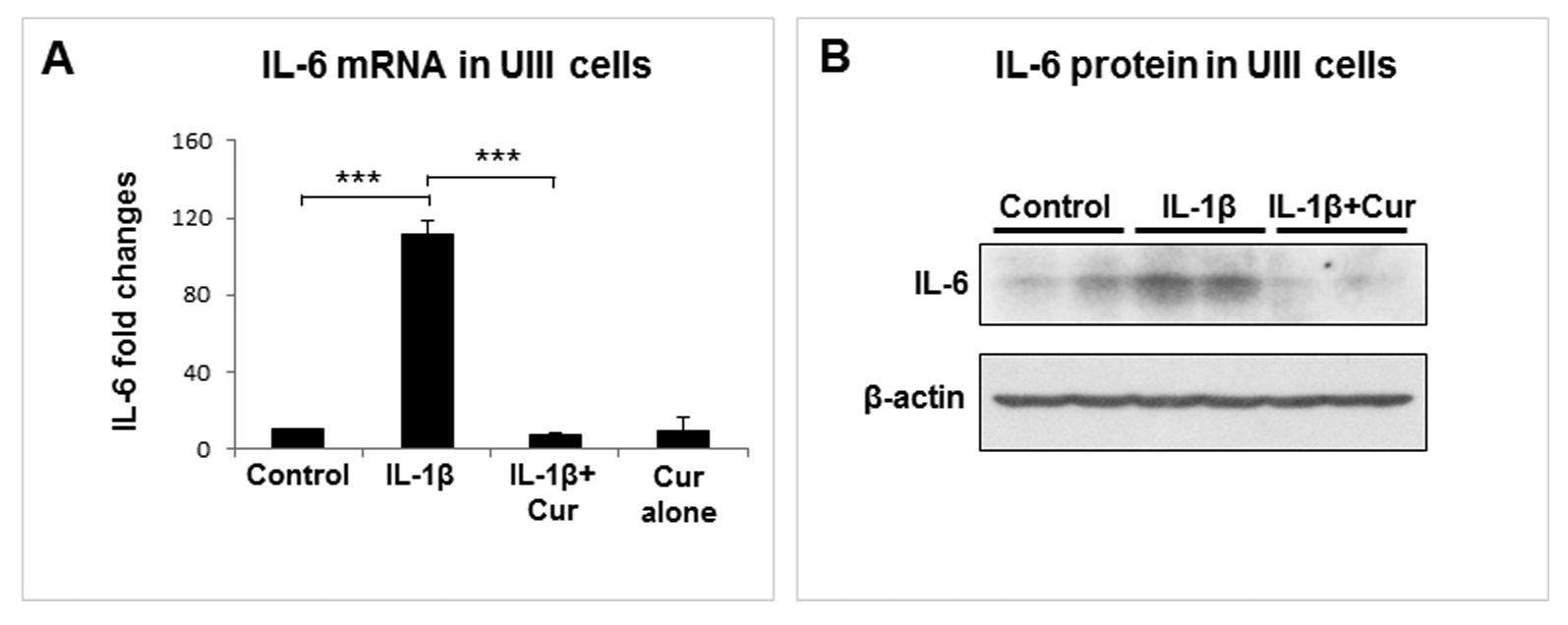

Supplement: S1 Fig — UIII cells were treated with vehicle (control), IL-1β (10ng/ml), or with a combination of IL-1β (10ng/ml) and curcumin (30μM) for 24hr. Expression of IL-6 was analyzed by qPCR (A) and Western blot (B). RPS17 expression was used as control for qPCR and β-actin was used as loading control for Western blot analysis. The values are expressed as the means ± S.E. (n = 3). ***, p < 0.001. (TIF) [file pone.0125627.s001.tif]

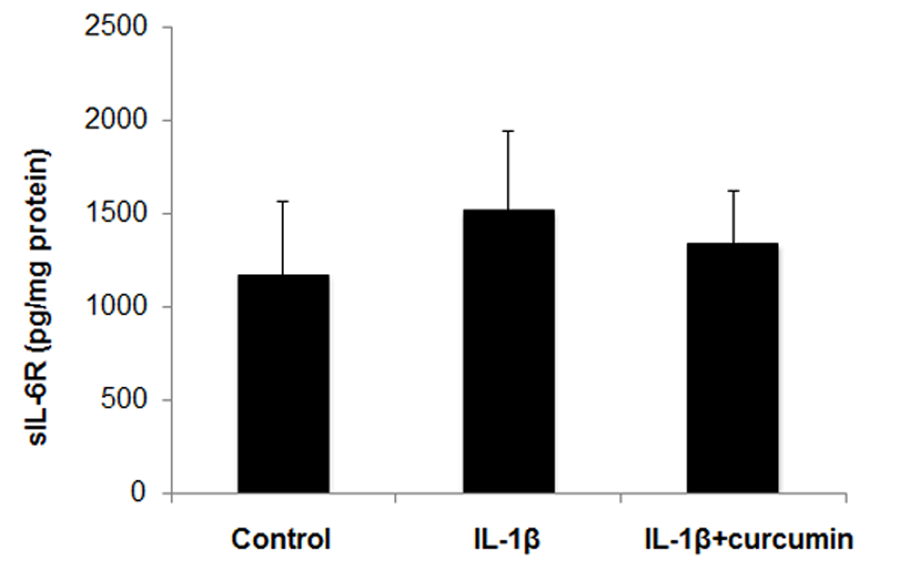

Supplement: S2 Fig — HuF cells were treated with either vehicle (control), IL-1β (10ng/ml), or with a combination of IL-1β (10ng/ml) and curcumin (30μM) for 24hr. The culture media were assessed for sIL-6R concentrations using a ELISA kit. The values were corrected for total protein and expressed as pg/mg protein and expressed as the means ± S.E. (n = 3). (TIF) [file pone.0125627.s002.tif]
