# Supplementary material for: Inhibition of IL-6 Signaling Pathway by Curcumin in Uterine Decidual Cells
Source: PLoS One. 2015 May 11;10(5):e0125627. doi: 10.1371/journal.pone.0125627 (PMC4427355; doi:10.1371/journal.pone.0125627)
Supplement: S1 Table — (DOCX) [file pone.0125627.s003.docx]

**S1 Table. Available clinical information of mothers/placentas**

|  | Control | Preterm |
| --- | --- | --- |
| Gestational age (wk) | 37 – 40 | 29 - 34 |
| Maternal age (yr)  Mean  Range | 29.8  22 - 37 | 29.6  21 - 34 |
| Parity  Mean  Range | 1.6  0 - 3 | 0.7  0 - 2 |
| Placental weight (g) | Not available | 405.5 |
| Cause of preterm birth | - | Unknown |
